# Genetic improvement of resistance to preharvest sprouting using a major QTL allele for embryo dormancy in rice

**DOI:** 10.1007/s11032-025-01623-8

**Published:** 2025-12-10

**Authors:** Kamal Bhattarai, Min Guo, Marya Bibi, Huayu Xu, Christian De Guzman, Xing-You Gu

**Affiliations:** 1 Agronomy, Horticulture, and Plant Science Department, South Dakota State University, Brookings, SD USA; 2 Agricultural College, Yangzhou University, Yangzhou, China; 3 Division of Agriculture, Rice Research and Extension Center, University of Arkansas, Stuttgart, AR USA; 4 Seed Molecular Biology Lab, South Dakota State University, Box 2140C, Brookings, SD 57007 USA

**Keywords:** Seed dormancy, Preharvest sprouting, Flowering time, Plant height, Quantitative trait locus, Genomic selection, Rice

## Abstract

Lack of seed dormancy (SD) often causes pre-harvest sprouting (PHS) or inadequate germination in cereal crops and seed production. This research aimed to integrate a dormancy (D) allele isolated from weedy rice at *qSD12* into the genetic background of a restoration-of-fertility line (RFL) to mitigate the PHS and germination problems. A hybrid F_2_ population was developed to map quantitative trait loci (QTLs) and model their epistatic and genotype-by-environment interactions. Twelve QTLs for flowering time (FT, 3), plant height (5) or SD (4) were identified. Of the 4 SD loci, *qSD12* explained most of the phenotypic variance (R^2^ = 0.34), and 2 (*qSD3* & *qSD6*) have the D alleles from the RFL. *qSD12* interacted with *qSD3* or *qSD7-2*, with the presence of the D allele at *qSD12* increasing the effects of the others, as well as the duration of seed storage at 24 or 40 ˚C for dormancy release. The F_2_ plant-derived F_4_ to F_6_ lines were marker-assisted selected. The major effect of *qSD12* on seed germination and on-panicle sprouting was confirmed with the F_4_ and F_5_ lines, respectively. A sample of F_2_ plant-derived backcross (BC_1_F_1_) plants, which are heterozygous for 2–4 of the SD QTLs, was evaluated for agronomic traits and genotyped with an array of SNP markers. Recurrent backcrossing with the RFL and genomic selection are used to pyramid the D alleles from *qSD12* and other locus/loci to develop new varieties and RF lines with improved resistance to PHS.

**Supplementary Information:**

The online version contains supplementary material available at 10.1007/s11032-025-01623-8.

## Introduction

Lack of seed dormancy frequently causes pre-harvest sprouting (PHS)-the germination on the plant before harvest under humid conditions in cereal crops. Recovery of the dormancy can improve crop resistance to PHS, but excessive dormancy promotes seed survival in the field, resulting in feral plants in the next seasons (Rodriguez et al. 2015; Mabry et al. 2023). Many quantitative trait loci (QTLs) have been associated with seed dormancy (SD) in barley (*Hordeum vulgare*), wheat (*Triticum aestivum*), rice (*Oryza sativa*), and sorghum (*Sorghum bicolor*) since the early 1990s (e.g., Ullrich et al. 1992; Anderson et al. 1993; Lin et al. 1998; Benech-Arnold and Rodríguez 2018). Allelic variants of the mapped QTLs can be selected to manipulate SD levels for crop varieties resistant to PHS (Nakamura, 2018; Sohn et al. 2021). Genetic factors considered for the selection include linkage drag, pleiotropy and epistasis of SD loci, as discussed in the previous research (Wang et al. 2020), and effect sizes of the QTLs in different genetic backgrounds.

A major QTL for SD is characterized by a large, constant effect on delay of germination in different populations and environments. Major QTLs reported for crop plants include *SD1* in barley (Ullrich et al. 1992; Hori et al. 2007), *Phs* (*Phs1*) and *QPhs.ocs-3 A.1* (*QPhs.pseru-3AS*) in wheat (Flintham 2000; Mori et al. 2005; Torada et al. 2005; Liu et al. 2013), and *qSD12* in rice (Gu et al. 2004; Zhang et al. 2017). In barley, *SD1* was delimited to a centromeric region likely containing at least 3 genes linked in coupling to enhance dormancy (Han et al. 1999), and a gene cloned from the QTL region encodes an alanine aminotransferase (Sato et al. 2016). In wheat, *QPhs.ocs-3 A.1/QPhs.pseru-3AS* was cloned as *TaMFT*, an ortholog of *MOTHER OF FT AND TFL1* in *Arabidopsis thaliana* (Nakamura et al. 2011; Liu et al. 2013). Both *Phs1* in wheat and *SD2* in barley were cloned as the *Mitogen-Activated Protein Kinase Kinase 3* genes (Nakamura et al. 2016; Torada et al. 2016). In rice, the dormancy allele at *qSD12* was identified from weedy accessions and delimited to a genomic region of < 100 Kb, which contains about 10 genes predicted to encode transcription factors or transposable elements (Gu et al. 2010). In addition to the main effects, a major QTL, such as *SD1* in barley and *qSD12* in rice, could also modify effects of the other QTLs by two or higher orders of epistasis to influence genotypic variation in SD (Ullrich et al. 1992; Gu et al. 2004). The dormancy and a non-dormancy allele at *qSD12* were introduced into the same genetic background, as a pair of isogenic lines (IL), where the QTL was associated specifically with embryo dormancy (Gu et al. 2008, 2015).

Rice varieties can be inbred or hybrid. A hybrid variety is the first filial (F_1_) generation of a hybrid between a male sterility (MS) and a restoration-of-fertility (RF) line and selected based on the degree of heterosis for yield (Virmani 1996). Hybrid rice has kept increasing in acreage in Central Southern United States for > 20 years because of its high yield potential (McBride et al. 2018). One of the technical challenges to the adoption of hybrid rice has been the “inadequate germination” of F_1_ seeds, such as < 70% at harvest (FCIC 2020). Hybrid seeds are developed on the MS plants whose panicles are partly enclosed within the leaf sheath. Thus, gibberellic acid (GA3) is applied to promote panicle emergence to increase seed setting rate by cross pollination (Virmani 1996). However, the GA application also stimulates germination during seed maturation, harvesting or transportation in humid environments, resulting in reduction in seed storability and vigor at sowing. Thus, we initiated a project to integrate SD genes into elite MS and RF lines to develop varieties that are resistant to PHS but insensitive to GA-induced on-plant germination.

This research consisted of three groups of experiments to address genetic or technical questions about *qSD12* in the genetic background of an RF line (RFL). The first group of experiments was based on a hybrid F_2_ population to map QTLs associated with SD and agronomic traits and to model epistasis between the QTLs or genotype-by-environment interactions for the dormancy release at different storage temperatures. The second group of experiments was based on the F_2_ plant-derived F_4_ and F_6_ lines to validate the major effect of *qSD12* on SD or seed sprouting rate on the panicles. And the third experiment started with a backcross (BC_1_F_1_s) to introduce a series of recombinants among the SD QTLs into the RFL background to manipulate the dormancy levels for new genotypes/varieties.

## Materials and methods

### Plant genotypes and cultivation

**Fig. 1:**
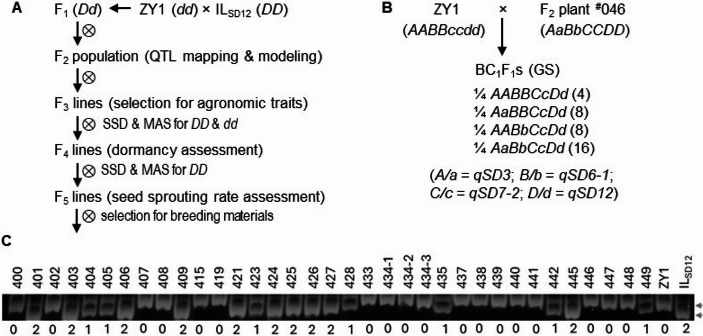
Breeding schemes for this research. **A.** A cross used to develop an F_2_ population and advanced lines. **B**. A backcross between an F_2_ plant and ZY1. **C**. A gel image showing marker genotypes of *qSD12* for the parental or F_2_-derived F_3_ plants. IL_SD12_ is a donor of the dormancy allele (*D*) at *qSD12.* ZY1 is a restoration-of-fertility line of hybrid rice. Genotypes and genotypic frequencies of the BC_1_F_1_s are listed, with the upper-/lower-case letters indicate the dormancy-enhancing/-reducing alleles at the 4 QTLs mapped for seed dormancy (SD) in the F_2_ population. Listed in the parentheses after the BC_1_F_1_ genotypes are the number of recombinant homozygotes for the heterozygous loci that can be isolated from their offsprings. The codes on the gel image indicate that the plant genotypes contain 0, I or 2 alleles at *qSD12* from the parent IL_SD12_, and the arrowheads in the right side indicate the polymorphic bands/alleles. SSD, single seed decent; MAS, marker-assisted selection for *qSD12*; and GS, genome-wide selection for marker genotypes

Two *indica*-type semidwarf lines, IL_SD12_ and Zhongyu No. 1 (ZY1), were selected as parents for the cross or backcrosses (Fig. 1A). IL_SD12_ is an isogenic line that contains the dormancy allele (*SD12*) at *qSD12* from SS18-2, an accession of weedy rice, in the genetic background of the early maturation line EM93-1 (*sd12*; Pipatpongpinyo et al. 2020). ZY1 is an elite variety or RF line developed by a collaboration between China and International Rice Research Institutes and was introduced from the USDA-ARS Genetic Stocks Oryza Collection (PI^#^ 614980). The ZY1/IL_SD12_ cross was used to develop an F_2_ population for QTL analysis. The F_2_ plants were selected to develop the F_3_ to F_5_ lines to purify genetic backgrounds of *qSD12* and evaluate its effects on seed dormancy in the F_4_ or seed sprouting rates on the panicles in the F_5_ generation.

To develop the mapping population, a seed sample from an F_1_ ZY1/IL_SD12_ plant was dried at the room temperature for > 6 months to break the dormancy before germination in a 30℃ incubator. The newly germinated seedlings were transplanted into pots (13.5 cm × 13.5 cm × 12 cm), with one plant per pot. The pots were filled with a mixture of clay soil and greenhouse medium (ProMix BX) in the 1:1 (v/v) ratio. These pots were assembled in water-tight containers (90 cm × 60 cm) on benches in a greenhouse. Day/night temperatures in the greenhouse were set at 28/22°C and the daylength was natural, except for the period from 45 to 75 d when a 10-h short-day treatment (8:00–18:00) was applied to synchronize flowering. The F_2_ plants were genotyped at the tillering stage and tagged for flowering dates when the first panicle of a plant emerged from the leaf sheath. Plant height was measured for the main tiller of a plant. Seeds were harvested at 40 d after flowering, air-dried in the greenhouse for 3 d, and then stored in a −20 °C freezer to maintain the status of primary dormancy and to equilibrate moistures of seed samples.

To purify genetic backgrounds of *qSD12*, 13 F_2_ plants were advanced to the F_3_ lines, and 4 plants from each of the 13 lines selected based on the marker genotypes (Fig. 1C) to develop F_4_ lines. The F_3_ and F_4_ lines were planted in field plots at Arkansas Rice Research & Extension Center in Stuttgart, AR. About 40 plants from a line were distributed in a plot, with the density being 15 cm between plants in a row and 20 cm between rows. Field management and fertilizer and herbicide applications were the same as the practices for breeding programs at the center. The field plots were checked for purity at the tillering and flowering stages to remove off-type or contaminated plants. Three to five plants from each of the F_4_ lines were harvested 40–45 d after flowering, and the seed samples were air-dried for 3 d before stored in a freezer for dormancy assessment.

Single plants that are homozygous for *SD12* were selected from different F_4_ lines and used to develop the F_5_ lines (Figs. 1A & C). The F_5_ and ZY1 plants were grown in a greenhouse to harvest intact panicles at 40 or 60 days after flowering (DAF) to evaluate seed sprouting rates.

An F_2_ plant (^#^046) that was homozygous for *SD12* was selected to backcross with ZY1 to develop the BC_1_F_1_ plants (Fig. 1B). The BC_1_F_1_ and ZY1 (control) plants were grown in a greenhouse, evaluated for seed dormancy and agronomic traits and genotyped for genomic selection of the SD loci and their genetic background.

### Seed dormancy assessment

Seed dormancy for the F_2_ and the F_2_-derived F_4_ and F_5_ lines was evaluated by germination testing. Preliminary testing was conducted using seed samples stored at the room temperature (23–24 ℃) for 0, 7 or 14 d to select a period (d) of after-ripening (DAR) that better displays the genotypic variation in germination percentage within a population or line. For a formal experiment, a sample of about 60 seeds was distributed on a piece of filter paper and imbibed with 8 ml water, three samples from a plant were prepared, and germination conditions were 30 ℃ and 100% relative humidity in an incubator set in dark condition. Germinated seeds (≥3 mm radicle protrusion) were counted at 3 (n_3_), 5 (n_5_) and 7 (n_7_) d after imbibition (DAI) to calculate germination percentage (GP) and index (GI) for a sample of N seeds:

1 $$\mathrm{GP}=100\times\left({\mathrm n}_3+{\mathrm n}_5+{\mathrm n}_7\right)/\mathrm N\left(\%\right)$$

2 $$\mathrm{GI}=100\times\left({\mathrm n}_3\times5+{\mathrm n}_5\times3+{\mathrm n}_7\times1\right)/\left(N\times7\right)(\%)$$

GP was used to measure the degree of primary dormancy immediately prior to the imbibition. GI, a weighted GP, was also used to quantify genotypic differences in germination velocity.

### Evaluation of seed sprouting rate on the panicle

The sprouting rate was evaluated for the eight F_5_ lines and ZY1 (control). Nine panicles for a line were selected from the plants flowered on the same day and harvested after 40 and 55 d. Immediately after each harvesting, nine panicles from each of the lines were distributed evenly on a double layer of moist regular weight brown germination papers (10′′×15′′; Anchor Paper Co., St. Paul, MN), covered with the other layer of germination paper wetted with a mist sprayer, and placed in an airtight light penetrance plastic container in a lab room for 7 d. The containers were monitored for relative humidity (RH) and temperature (23.3 ± 0.3˚C) using a data logger (LogTaq^®^), the RH level was checked every 12 h, and mist spraying was applied, if necessary, to keep a highly humid germination environment. After 7 d, each of the panicles was counted for the numbers of seeds (filled spikelet) sprouted (N_s_; visible radicle and/or coleoptile) and non-sprouted (N_ns_) to calculate the sprouting rate (SR):

3 $$\mathrm{SR}=100\times{\mathrm N}_{\mathrm s}/({\mathrm N}_{\mathrm s}+{\mathrm N}_{\mathrm{ns}})\;(\%)$$

### Regular and high-throughput genotyping

The F_2_ to F_5_, BC_1_F_1_ and parental plants were genotyped with simple sequence repeat (SSR) or short insertion/deletion (InDel) markers located within the *qSD12* locus. Genomic DNAs were extracted from fresh leaves of 4-week seedlings using CTAB method (Doyle and Doyle 1987). Primers for polymerase chain reaction (PCR) and procedures for PCR and gel electrophoresis to amplify or display the marker alleles were the same as previously described in Pipatpongpinyo et al. (2020).

The F_2_, BC_1_F_1_ and parental plants were also genotyped with the single nucleotide polymorphism (SNP) array 1k-RiCA (Arbelaez et al. 2019). DNA samples were quantified using nano-drop spectrophotometry (ND2000, Thermo Fisher Sci., USA). The amount of 100 µL at 30 ng DNA/µL from a plant was used for SNP genotyping (AgriPlex Genomics, Cleveland, USA). The array consists of 1076 loci (1059 single base substitutions, 5 single base deletions & 12 InDels of 2–13 bases) distributed on the 12 chromosomes. The gross success rate was 92.3% in the genotyping experiment.

### Linkage map construction and QTL mapping

Genotyping data from a subpopulation of 187 F_2_ plants were used to construct a linkage map. Of the 1076 markers genotyped for the population, 986 had fail rates of 0% (770), 1–9.9.9% (181) or 10–29% (35). Of the 986 markers, 361 (36.6%) were polymorphic between the parents and were used to develop a linkage map. The map was constructed using a mapping program of the QTL IciMapping (V4.2) software (Meng et al. 2015), with the LOD score set at 3 and the genetic distance in centiMorgan (cM) converted with Kosambi mapping function. Linkage groups/chromosomes of the map were plotted using MapChart software V.2.32 (Voorrips 2002).

The linkage map and marker genotyping data from the F_2_ population were used to identify QTLs associated with flowering time (*qFT*, evaluated by days to flowering), plant height (*qPH*), or seed dormancy (*qSD*), which were quantified by the mean GP and GI of three replicates for a plant. A composite interval mapping program of the QTL IciMapping software was used to locate the QTLs and to estimate their effects and heritability (R^2^).

### Modeling of QTL additive effects

Additive genetic effects of multiple loci for such a quantitative trait seed dormancy could include their additive main and epistatic components, which cumulative and can be fixed by selective breeding. Considering the size of the F_2_ population and the map resolution, all the QTLs mapped for each of the traits were combined in a linear regression model to estimate for their additive (*a*) and *a*×*a* interactional effects,

4 $$y_l=\mu+{\textstyle\sum_{}a_ix_i+\sum_{}}a_ia_jw_{ij}+\varepsilon_l$$

where, *y*_*l*_ is the dependent variable for the phenotypic value of plant *l*; *µ* is the model mean; *x*_*i*_s are independent variables for the additive component of locus *i* (*i* = 1, 2 … n, the number of QTLs detected in the population), with *x* coded as −1 for the ZY1-like homozygote, 0 for heterozygote, and 1 for IL_SD12_-like homozygote; *a*_*i*_ is the additive effect of locus *i*; *w*_*ij*_s are the independent variables for the epistasis between loci *i* and *j*, (*i* < *j* ≤ n), with *w* coded as the product of dummy variables for *x*_*i*_ and *x*_*j*_; *a*_*i*_*a*_*j*_ is the effect of *a*×*a* interaction; and *ε*_*j*_ is the error term of the model.

### Dormancy-breaking treatments and their effect Estimation

Two treatments, after-ripening (AR) and heating, were used to break seed dormancy associated with *qSD12*. Seeds from the 192 F_2_ plants were bulked based on the *DD*, *Dd* and *dd* genotypes for the dormancy-enhancing (*D*) or -reducing (*d*) alleles at *qSD12*. The three genotypes of seeds were moved from the freezer to the room temperature (23–24 ˚C) for 3, 5 or 7 weeks for AR treatment, or to a 40–42˚C oven for 1, 2 or 3 weeks for heating treatment. Seeds sampled at each of the time points were germinated, with 27 samples/genotypes for the AR or 14 samples/genotype for the heating treatment, using the above-stated methods.

Main and interactional effects of the genotypes and treatments were estimated using the linear regression model,

5 $$y_l=\mu+ax_i+dz_i+\tau x_j+i_{\tau a}w_{1ij}+i_{\tau d}w_{2ij}+\varepsilon_l$$

where, *y*_*l*_ is the dependent variable for the GP (Eq. 1) or GI (Eq. 2) of sample *l*; *µ* is the model mean; *x*_*i*_ is the independent variable for the additive component of *qSD12*, with *i*=−1, 0 and 1 for the *dd*, *Dd* and *DD* genotypes, respectively; *a* is the regression coefficient or the additive effect; *z*_*i*_ is the variable for *qSD12*’s dominance component, with *i*=−0.5 for the *dd* or *DD* genotypes or 0.5 for the *Dd* genotype; *d* is the dominance effect; *x*_*j*_ is the independent variable for the main effect of the treatment, with *j*=−1, 0 and 1 for the 3, 5 7 weeks, respectively, in the AR treatment or for 1, 2 and 3 weeks, respectively, in the heating treatment; *τ* is the main effect of the treatment; *w*_*1ij*_ and *w*_*2ij*_ are variables for interactions of the treatment with the additive and dominance components, respectively, and coded as the product of *i* and *j*; *i*_*τa*_ and *i*_*τd*_ are the interactional effects; and *ε*_*j*_ is the error term of the model. Linear regression analysis was performed using the SAS GLM or REG programs (SAS Institute 2020). A stepwise selection at a significant level of 5% was used to retain component variables in the models.

## Results

### Parental differences and segregation patterns in the F_2_ population

**Fig. 2:**
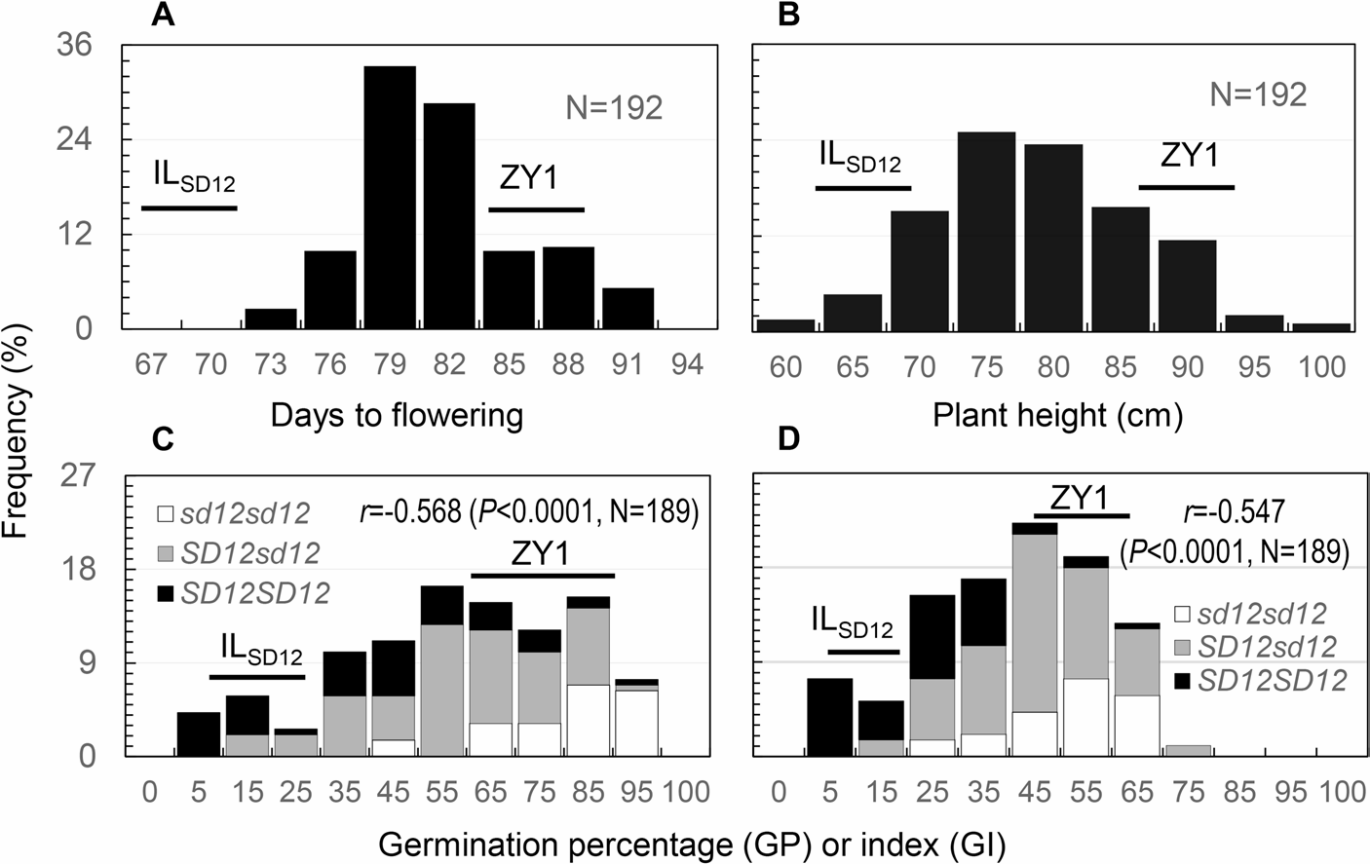
Frequency distributions of flowering time (**A**), plant height (**B**) and seed dormancy (**C** & **D**) in the F_2_ population. The population was developed from a cross between IL_SD12_ and ZY1. Seed dormancy was evaluated by germination percentage (**C**) and index (**D**). Columns indicate frequencies for the N number of plants. Line segments indicate ranges of the parents for the trait measurements. The negative correlation coefficients (r) indicate that the GP and GI values decreased with the number of the dormancy-enhancing allele (*SD12*) at *qSD12*

The donor (IL_SD12_) and recipient (ZY1) parents differed not only in SD but also in flowering time (FT) and plant height (PH). ZY1 flowered ~ 20 d later and were ~ 25 cm taller than IL_SD12_ (Figs. 2A & B). The differences are like those between the parental lines of hybrid varieties used for rice production. A range of variation was observed for each of the traits in the F_2_ population. All the traits displayed a certain degree of transgressive segregation for one (FT & SD) or two (PH) directions, as shown by their frequency distributions in the population (Fig. 2). The segregation patterns suggest that the donor and recipient parents differentiate at multiple loci for each of the traits and both carry effect-increasing and -decreasing alleles at the loci. The loci differentiated for SD include *qSD12*, as its genotypes were strongly correlated with GP and GI (*r*=−0.55 or 0.57; *P* < 0.0001) (Figs 0.2 C and D).

### A high-resolution linkage map

A linkage map was constructed using a subpopulation of 187 F_2_ plants genotyped for 343 SNP markers on the 12 chromosomes (Figure S1). The map length is 1329 cM, with the mean inter-marker distance being 4.0 (± 0.82) cM. There are four large intervals of 34 to 46 cM on chromosomes 6, 7, 10 or 11, suggesting that the two parents lack polymorphism for the genomic regions. Segregation distortion loci were detected on chromosomes 3 (SDL3) and 5 (SDL5). Frequencies for the alleles from ZY1 were 0.446 at SDL3 (χ^2^ = 12.1) or 0.538 at SDL5 (χ^2^ = 8.3), suggesting that genes causing the distortion are from both parents.

### QTLs associated with flowering time, plant height or seed dormancy

**Fig. 3:**
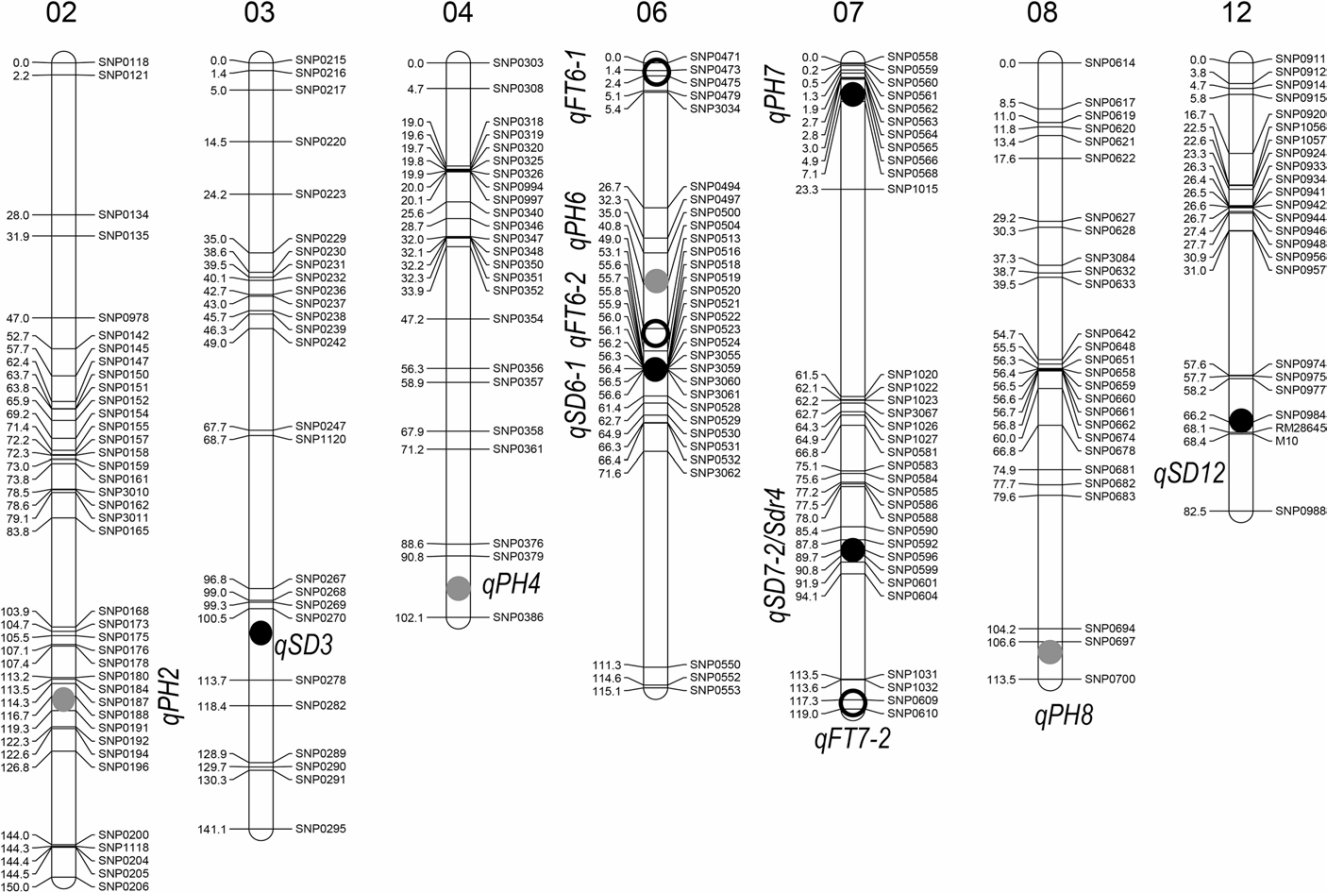
Map positions of QTLs for seed dormancy (qSD), flowering time (qFT) and plant height (qPH) detected in the F_2_ ZY1/IL_SD12_ population. The map was constructed based on 187 F_2_ plants (374 gametes) and adopted from Fig. S1. Circles indicate the QTL peak positions on the likelihood distributions (Fig. S2)

A total of 12 QTLs for flowering time (*qFT6-1*, *6 − 2* & *7 − 2*), plant height (*qPH2*, *4*, *6*, *7* & *8*) or seed dormancy (*qSD3*, *6 − 1*, *7 − 2* & *12*) were detected in the F_2_ population (Figure S2). The QTLs are mapped on 7 chromosomes (chr), including 4 on chr 6 and 3 on chr 7 (Fig. 3). Based on the map positions, *qFT7-2* (Gu & Foley, 2007) and one or more of the 4 SD loci, such as *qSD3* (Ye et al. 2010) and *qSD6-1* and *12* (Gu et al. 2004; Zhang et al. 2017), were previously detected. Table S1 summarized some parameters of the 12 loci, including additive (*a*) and dominance (*d*) effects, proportion of the phenotypic variance explained (R^2^), and the parental source of the effect-increasing or reducing alleles. The mapped QTLs varied in the effect size and R^2^. For example, *qFT6-2* (41%), *qPH4* (23%), and *qSD12* (32/36%) accounted for most of the variances for DTF, PH and GP/GI, respectively, in the population. Both parents contributed the effect-increasing or -reducing alleles to at least one of the QTLs for each of the traits. For example, IL_SD12_ contributed the dormancy (D) alleles to *qSD12* and *qSD7-2*, while ZY1 donated the D alleles to *qSD3* and *qSD6-1*. The allelic distribution patterns explain the transgression segregations for the three traits in the F_2_ population (Fig. 2).

**Fig. 4:**
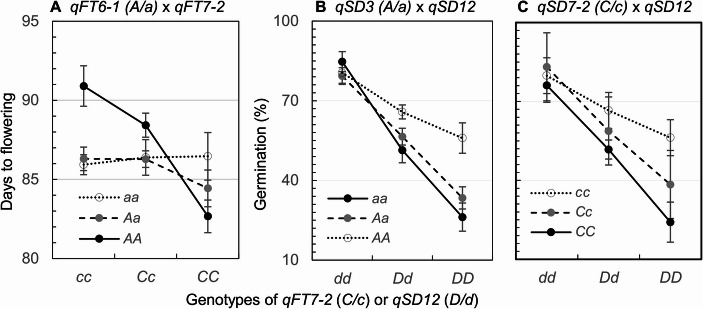
Patterns of Interactions between QTLs for flowering time (*qFT*; **A**) or seed dormancy (*qSD;* **B** & **C)** in the F_2_ population. The lower- and upper-case letters in the parentheses indicate the alleles from the ZY1 and IL_SD12_ parents, respectively. Circles (bars) indicate genotypic means (s.e.)

Modeling analysis (Eq. 4) confirmed additive effects of the 3 FT, 5 PH or 4 SD QTLs and detected three sets of *a*×*a* epistasis for FT or SD. The two types of genic effects together accounted for 37%, 46% and 62% of the variances for DTF, PH and GP, respectively, in the F_2_ population (Table 1). One of the epistatic interactions occurred between *qFT6-1* and *qFT7-2*. The allele at *qFT6-1* from the parent IL_SD12_ delayed or promoted flowering when the genotypes are homozygous for the alleles at *qFT7-2* from ZY1 and IL_SD12_, respectively (Fig. 4A). The other interactions occurred between *qSD12* and *qSD3* or *qSD7-2* and displayed a similar pattern. Variation in GP among the three *qSD3* or *qSD7-2* genotypes increased with the copy number of *SD12* in the genetic background (Figs. 4B & C), suggesting that *SD12* may regulate dormancy genes at the other loci.

**Table 1:** Summary of additive (*a*) and *a*×*a* (*i*_*aa*_) effects of the QTLs associated with flowering time (qFT), plant height (qPH) or seed dormancy (qSD) in the F_2_ ZY1/IL_SD12_ population

Component	Effect	S.E.	Probability	*R*^2^ (%)
Flowering time evaluated by days to flowering				
*µ*	86.31	0.25	< 0.0001	
*a*_*1*_ (*qFT6-1*)	0.99	0.37	0.0076	3.0
*a*_*2*_ (*qFT6-2*)	−3.23	0.37	< 0.0001	29.4
*a*_*3*_ (*qFT7-2*)	−1.15	0.39	0.0033	2.2
*i*_*a1a3*_	−1.35	0.52	0.0095	2.4
Total				37.0
Plant height in cm				
*µ*	77.96	0.45	< 0.0001	
*a*_*1*_ (*qPH2*)	2.55	0.64	0.0001	4.4
*a*_*2*_ (*qPH4*)	−5.27	0.65	< 0.0001	24.7
*a*_*3*_ (*qPH6*)	−2.94	0.63	< 0.0001	5.7
*a*_*4*_ (*qPH7*)	−3.60	0.62	< 0.0001	6.8
*a*_*5*_ (*qPH8*)	−2.31	0.63	0.0004	4.0
Total				45.6
Seed dormancy evaluated by germination percentage (%)				
*µ*	59.03	1.15	< 0.0001	
*a*_*1*_ (*qSD3*)	6.69	1.49	< 0.0001	5.6
*a*_*2*_ (*qSD6-1*)	11.50	1.67	< 0.0001	11.0
*a*_*3*_ (*qSD7-2/Sdr4*)	−8.46	1.74	< 0.0001	6.3
*a*_*4*_ (*qSD12*)	−21.04	1.62	< 0.0001	34.4
*i*_*a1a4*_	8.48	2.11	< 0.0001	3.4
*i*_*a3a4*_	−5.11	2.46	0.0395	0.9
Total				61.6

The model mean (*µ*), additive (*a*) and epistatic (*i*_*aa*_) effects, standard error (S.E.), F-test probability, and proportion of the variance explained by the component or model (R^2^) were estimated using Eq. 4. A positive/negative *a* value indicates that the allele from the parent IL_SD12_ increased/decreased the effect value

### Genotypic differences of *qSD12* in response to after-ripening or heating treatments

**Fig. 5:**
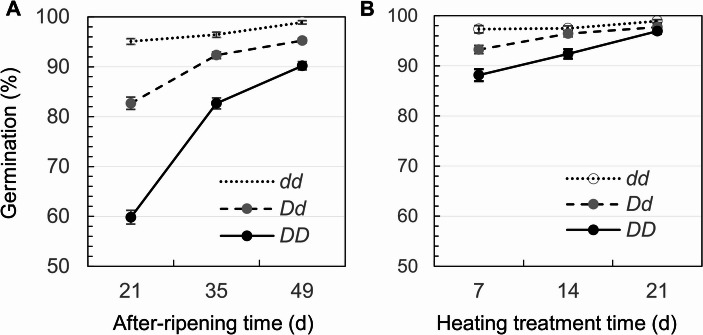
Genotypic differences of *qSD12* (*D/d*) for the release of seed dormancy in the after-ripening (**A**) or heating (**B**) trials. The dormancy was evaluated by germination percentages for seed samples from the three genotypes of F_2_ plants and treated with the indicated times. The circles (bars) indicate the means (s.e.) of 27 (**A**) or 14 (**B**) samples

A large difference for dormancy release between the AR and heating treatments was observed. Seed samples from the three genotypes of *qSD12* reach a similar level (88–99%) of germination after 1 week of the heating or 7 weeks of the AR treatment, and 3 weeks of the heating treatment broke > 97% of the dormancy in the samples (Fig. 5). Thus, heating treatment is more efficient to break SD than AR at 24˚C, and the temperature of 40 ˚C has little negative effect on seed viability and germination.

The model mean (*µ*), additive (*a*) and epistatic (*i*_*aa*_) effects, standard error (S.E.), F-test probability, and proportion of the variance explained by the component or model (R^2^) were estimated using Eq. 4. A positive/negative *a* value indicates that the allele from the parent IL_SD12_ increased/decreased the effect value.

The germination level for each of the *qSD12* genotypes increased with times of the AR/heating treatments (Fig.5) because of the dormancy release. Modeling (Eq. 5) analysis detected additive (*a*) and dominance (*d*) effects of *qSD12*, main effect (*τ*) of the times, and their interactions (*i*_*τa*_ & *i*_*τd*_), with the additive component contributed most to the total variances in GP in the AR experiment (Table 2). The *a*, *τ* and *i*_*τa*_ effects on GP were significant, and the *a* and *τ* components accounted for a similar level (28% vs. 23%) of the variance for GP in the heating experiment (Table 2). The G-by-E interactions explained the non-linear increase in GP with treatment times in the experiments (Fig. 5).

### Effects of *qSD12* on seed dormancy and sprouting rate in the F_4_ or F_5_ lines

**Fig. 6:**
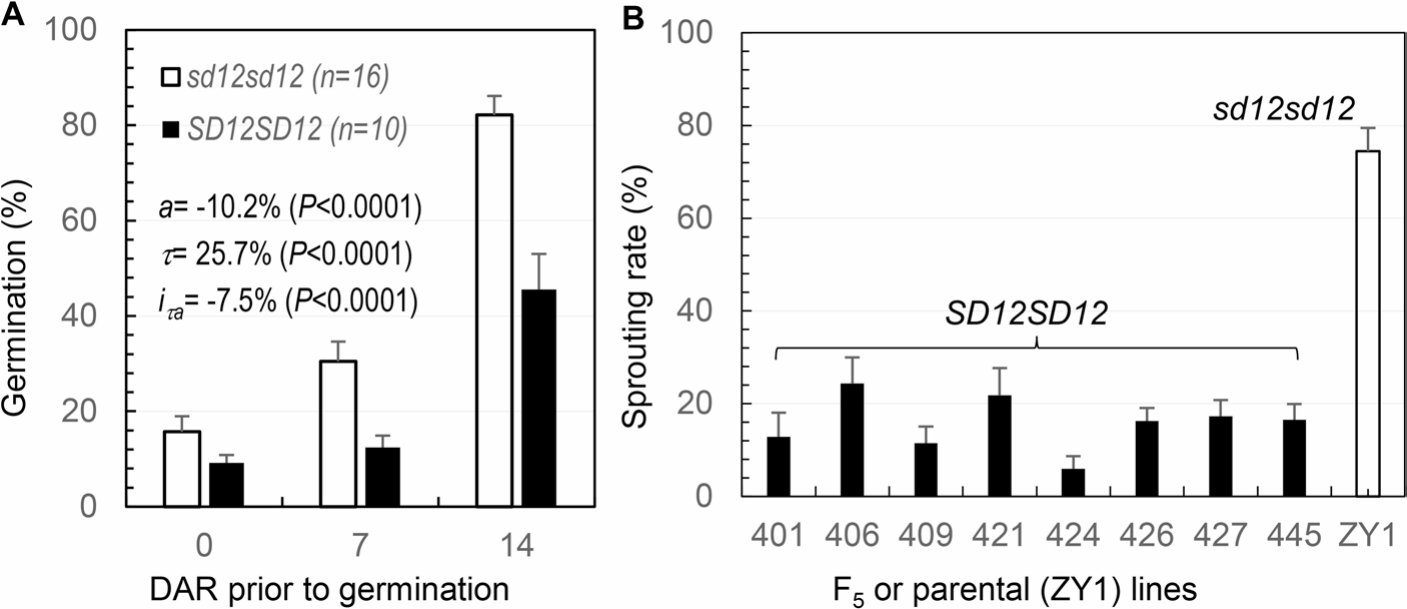
Genotypic differences in seed dormancy or sprouting rate. **(A)** Seed dormancy evaluated by germination percentages for the F_4_ lines. Seed samples were harvested from the two genotypes of F_4_ lines grown in a field environment. Columns (bars) indicate the means (s.e.) of the indicated (n) lines. **(B)** Seed sprouting rates on the panicles from the F_5_ or parental (ZY1) lines. The F_5_ lines (401–445) were homozygous for *SD12*. The panicles were harvested from greenhouse plants at 60 d after flowering. Columns (bars) indicate mean (s.e.) sprouting rates of 9 panicles

A total of 28 F_4_ lines were homozygous for the dormancy (*D*) and non-dormancy (*d*) alleles at *qSD12* and showed no segregation for agronomic traits in the field environment. Mean difference in GP between the *DD* and *dd* homozygotes was 6%, 18% and 37% at 0, 7 and 14 DAR, respectively (Fig. 6A). Both additive (*a*=−10%) and additive-by-AR time (*i*_*τa*_=−8%) effects acted to reduce germination or maintain SD (Fig. 6A). The results confirmed the major effect of *qSD12* in the advanced generation under the field conditions.

**Table 2:** Summary of component effects of *qSD12* on germination percentage after different periods of after-ripening (24 ˚C) or heating (40 ˚C) treatment

Component	After-ripening for 3, 5 or 7 weeks			Heating for 1, 2 or 3 weeks		
	Effect (%)	Probability	*R*^2^ (%)	Effect (%)	Probability	*R*^2^ (%)
*µ*	88.6	< 0.0001	-	95.4	< 0.0001	-
*A*	−9.6	< 0.0001	41.4	−2.7	< 0.0001	27.5
*D*	2.9	< 0.0001	1.3	-	-	-
*τ*	7.4	< 0.0001	27.2	2.5	< 0.0001	23.2
*i*_*τa*_	6.6	< 0.0001	13.1	1.8	< 0.0001	8.0
*i*_*τd*_	−2.3	0.0076	0.5	-	-	-
Total			83.4			58.7

The mean (*µ*), additive (*a*), dominance (*d*), treatment (*τ*) and interactional (*i*) effects, F-test probability and proportion of the variance explained by the component or model (R^2^) were estimated using Eq. 5. A positive/negative *a* value indicates that the allele from th, e parent IL_SD12_ increased/decreased germination.

The seed sprouting rate on the panicles sampled at 40 DAF was low (< 5%) and there was no difference between the F_5_ and parental (ZY1) lines. A large difference was observed for the panicles harvested at 55 DAF (Fig. 6B). The 8 F_5_ lines varied in SR from 6 to 25%, while the parental control had ~ 75% seeds germinated on the panicles. The F_5_ and ZY1 lines were homozygous for the *D* and *d* alleles, respectively (Fig. 1C). Thus, the observed difference in SR at 55 DAF was mainly the effect of *qSD12*.

### Genomic differences among the F_2_ plant-derived BC_1_F_1_ plants

The F_2_ plant ^#^046 was a strongly dormant genotype selected from the low end (10% germination) of the frequency distribution (Fig. 2C). This plant carries 49.3% alleles from the parent ZY1 at 342 polymorphic loci on the genome, with 19.9% of the loci homozygous for the ZY1 alleles (Fig. S3A). Based on genotypes for the loci, the plant is heterozygous for *qSD3* (*A/a*) and *qSD6-1* (*B/b*) but homozygous for the D alleles at *qSD7-2* (*C/c*) and *qSD12* (*D/d*) from the parent IL_SD12_. Thus, the plant genotype, designated *AaBbCCDD*, assembled the D alleles at the 4 loci from both ZY1 (*AABBccdd*) and IL_SD12_ (*aabbCCDD*).

**Fig. 7:**
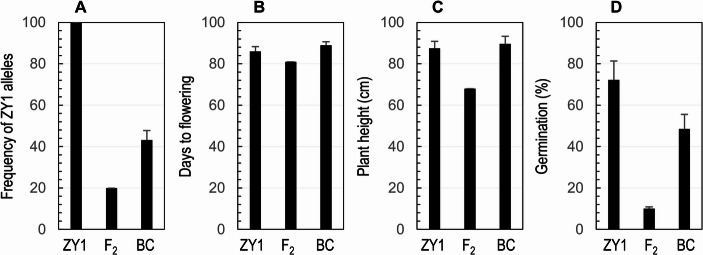
Differences among the ZY1, F2 #046 (F2) and BC1F1 (BC) plants for allelic frequency (**A**), time to flowering (**B**), plant height (**C**) and seed dormancy (**D**). The frequency was estimated for the alleles from ZY1 based on 342 SNP loci. Seed dormancy was evaluated by germination percentage. The columns (bar) are means (s.d.) of 12 BC_1_F_1_ or 10 ZY1 plants

Twelve BC_1_F_1_ plants were genotyped with the SNP array, which varied in genotypic frequency for the ZY1-like homozygotes at 342 loci from 34.7% to 49.2% in the BC_1_F_1_ plant ^#^6 (Fig. S3B). On average, the BC_1_F_1_s have 43.1% of their genomes synchronized by the ZY1 genome (Fig. 7A). Both FT and PH were similar between the BC_1_F_1_ and ZY1 plants (Figs. 7B & C). Whereas the germination rate of non-after-ripened seeds was lower for the BC_1_F_1_s (mean 48%; varying from 39% to 58%) than for ZY1 (72%) (Fig. 7D). The BC_1_F_1_ plants consisted of four genotypes that are heterogeneous for 2, 3 or 4 of the QTLs for SD (Fig. 1B). Thus, the observed difference (24%) in germination rate could be explained by main and epistatic effects from all the four QTLs, and a series of non-allelic recombinants, such as homozygotes for the D allele(s) at 1 to 4 of the loci, can be isolated from progenies of the BC_1_F_1_s (Fig. 1B) to manipulate genotypic variation in the dormancy degree.

## Discussion

The three groups of experiments conducted in this research confirmed the major effect of *qSD12* and developed plant genotypes and lines of new information required to integrate the QTL allele into inbred and hybrid varieties. The major effect was evaluated for seed dormancy and on-panicle sprouting rates in different generations/genetic backgrounds in controlled and/or field environments (Table 1; Fig. 6). The genotypes include those selected from the F_5_ lines that are homozygous for the D allele of *qSD12* and from the BC_1_F_1_ plants genotyped with the SNP array (Fig. 7). The information includes the high-resolution linkage map (Fig. S1), genetic parameters evaluated for the FT, PH or SD QTLs in the background of the RF line (Table 1), and technical parameters for the dormancy release in the two storage temperatures (Fig. 5).

Additive gene effects are inheritable across generations and can be selected for inbred and hybrid varieties to mitigate the PHS problem. The additive effect of *qSD12* (34%) contributed more to the total variance than the summation of the effects of the other 3 loci (22%) in the population (Table 1). Based on the size of effects on reduction in germination and sprouting rates (Fig. 6) and the association with embryo dormancy (Gu et al. 2015), the D allele of *qSD12* is idea for improving the resistance of inbred and hybrid varieties to on-plant germination.

Two QTLs, *Sdr4* (Lin et al. 1998) and *qSD7-2* (Gu et al. 2004), were mapped to a genomic region on the long arm of chr 7. *Sdr4* is 1032 bp in the genomic DNA sequence, which encodes a protein unknown for molecular function (Sugimoto et al. 2010). Fine-mapping of the genomic region genetically dissected *qSD7-2* from *Sdr4* and revealed a pleiotropic effect of *qSD7-2* on plant height (Ye et al. 2013). It is likely the SD QTL mapped in this population is *Sdr4*, as it has no effect on PH. DNA sequences for this gene and promoter regions from the parents can be used to confirm the hypothesis and to identify functional mutation(s).

Introduction of the D allele at *qSD12* into a new genetic background could alter the effect size or direction of the other SD gene(s) through epistatic interactions. For example, the D allele of *qSD3* acted to increase and decrease germination in the absence and presence of the D allele at *qSD12*, respectively (Fig. 4B). The effect of *qSD7-2/Sdr4* varied greatly with the copy number of the D allele at *qSD12* (Fig. 4C). The epistasis suggests that *qSD12* is also involved in regulation of the other genes for SD (Gu et al. 2004). The BC_1_F_1_ plant-derived BC_1_F_2_s are selected to model two or higher orders of epistasis. Similar patterns of epistasis may also occur to major QTLs reported for SD in the other cereal crops (Mori et al. 2005; Torada et al. 2005; Hori et al. 2007; Liu et al. 2013). Further research is needed to elucidate regulatory mechanisms of the QTL underlying genes for the development or release of SD (Nakamura et al. 2011; Liu et al. 2013; Sato et al. 2016; Torada et al. 2016).

Hybrid breeding to improve the resistance to PHS required generations of selection for SD loci and their genetic backgrounds. Marker-assisted selection (Fig. 1C) was used to develop the F_5_ lines, which are homozygous the D allele at *qSD12* and have the agronomic traits like the RF line ZY1. Three F_6_ lines homozygous for the D allele are entered into a breeding program to test its effect on germinability of hybrid F_1_ seeds. Some F_6_ lines from the F_5_ plants are being used to pollinate MS lines to test fertility restorability, germinability of hybrid seeds and combilities in a breeding program. An SNP array-based genomic selection (Fig. S3) was used to identify the BC_1_F_1_ genotypes (Fig. 1B) for recurrent backcrossing with ZY1. The SNP genotyping data from the F_2_ and BC_1_F_1_ plants (Fig. 7) suggest that the number of backcross generations used to synchronize the genetic background with the recurrent parent is mainly determined by the selected plants for recurrent backcrossing. We are advancing the backcross and genomic selection to combine the D alleles at the *qSD12* and other SD locus/loci in the same genotypes to select new varieties or RF lines with improved resistance to PHS or on-plant germination.

## Supplementary Information

Below is the link to the electronic supplementary material.

Supplementary Material 1 (DOCX 1.55 MB)

Supplementary Material 2 (DOCX 782 KB)

## Data Availability

Data are available upon request.
