# Supplementary material for: Genetic improvement of resistance to preharvest sprouting using a major QTL allele for embryo dormancy in rice
Source: Mol Breed. 2025 Dec 10;45(12):99. doi: 10.1007/s11032-025-01623-8 (PMC12696218; doi:10.1007/s11032-025-01623-8)
Supplement: Supplementary file 1 — Supplementary Material 1 (DOCX 1.55 MB) [file 11032_2025_1623_MOESM1_ESM.docx]

**Genetic Improvement of Resistance to Preharvest Sprouting using a Major QTL Allele for Embryo Dormancy in Rice**

Kamal Bhattarai,^1^ Min Guo, ^1,2^ Marya Bibi,^1^ Huayu Xu,^1^ Christian De Guzman,^3^ Xing-You Gu^1^

**Supplementary Information Figure S1**

**Supplementary Information Figure S2**

**Supplementary Information Figure S3**

**Supplementary Information Table S1**

**Table S1**. Summary of parameters for flowering time (*qFT*), plant height (*qPH*) and seed dormancy (*qSD*) QTLs detected in the F_2_ ZY1/IL_SD12_ population.

| QTL | Chr | Peak (cM) | Marker | LOD | *a* | *d* | R^2^ (%) | Donor |
| --- | --- | --- | --- | --- | --- | --- | --- | --- |
| *qFT6-1* | 6 | 2 | SNP0475 | 3.6 | 1.3 | -0.9 | 4.5 | IL_SD12_ |
| *qFT6-2* | 6 | 51 | SNP0513 | 25.2 | -3.5 | -2.9 | 41.5 | ZY1 |
| *qFT7-2* | 7 | 119 | SNP0609 | 3.5 | -1.3 | 0.8 | 4.5 | ZY1 |
| *qPH2* | 2 | 117 | SNP0188 | 5.06 | 2.98 | 1.03 | 6.5 | IL_SD12_ |
| *qPH4* | 4 | 98 | SNP0386 | 15.0 | -5.3 | -2.0 | 23.0 | ZY1 |
| *qPH6* | 6 | 41 | SNP0504 | 4.9 | -2.9 | -0.3 | 6.1 | ZY1 |
| *qPH7* | 7 | 4 | SNP0566 | 8.4 | -3.9 | -0.1 | 11.1 | ZY1 |
| *qPH8* | 8 | 107 | SNP0697 | 3.2 | -2.3 | 0.2 | 3.9 | ZY1 |
| *qSD3* | 3 | 104 | SNP0270 | 5.4 (GP) | 7.8 | -2.5 | 6.3 | ZY1 |
|  |  |  |  | 5.3 (GI) | 5.8 | -1.0 | 6.5 |  |
| *qSD6-1* | 6 | 57 | SNP0518 | 9.9 (GP) | 12.0 | 0.3 | 11.1 | ZY1 |
|  |  |  |  | 10.0 (GI) | 8.9 | 0.1 | 11.5 |  |
| *qSD7-2* | 7 | 90 | SNP0592 | 6.5 (GP) | -9.6 | -2.8 | 6.7 | IL_SD12_ |
| (*Sdr4*) |  |  |  | 4.3 (GI) | -5.4 | -0.6 | 4.3 |  |
| *qSD12* | 12 | 66 | SNP0984 | 24.4 (GP) | -20.0 | -2.3 | 32.3 | IL_SD12_ |
|  |  |  |  | 26.7 (GI) | -15.3 | -2.7 | 36.2 |  |

Note: The QTL LOD values and map/peak positions on the chromosomes (Chr) are also shown in Figure S3. Seed dormancy was evaluated by germination percentage (GP; Eq. 1) and index (GI; Eq. 2). The QTL additive (*a*) and dominance (*d*) effects and heritability (R^2^) were estimated by a composition interval mapping program. Donor indicates that the QTL alleles from the IL_SD12_ or ZY1 parent are responsible for the positive or negative additive values.
