# Supplementary material for: Genetic improvement of resistance to preharvest sprouting using a major QTL allele for embryo dormancy in rice
Source: Mol Breed. 2025 Dec 10;45(12):99. doi: 10.1007/s11032-025-01623-8 (PMC12696218; doi:10.1007/s11032-025-01623-8)
Supplement: Supplementary file 2 — Supplementary Material 2 (DOCX 782 KB) [file 11032_2025_1623_MOESM2_ESM.docx]

Manuscript Title: Genetic Improvement of Resistance to Preharvest Sprouting using a Major QTL Allele for Embryo Dormancy in Rice
Manuscript ID: a7a2a321-105b-4323-bfb6-26382aed7e1c v1.0

Query #2. Please provide the full uncropped Gels and Blots image(s) as file type ‘supplementary file’.


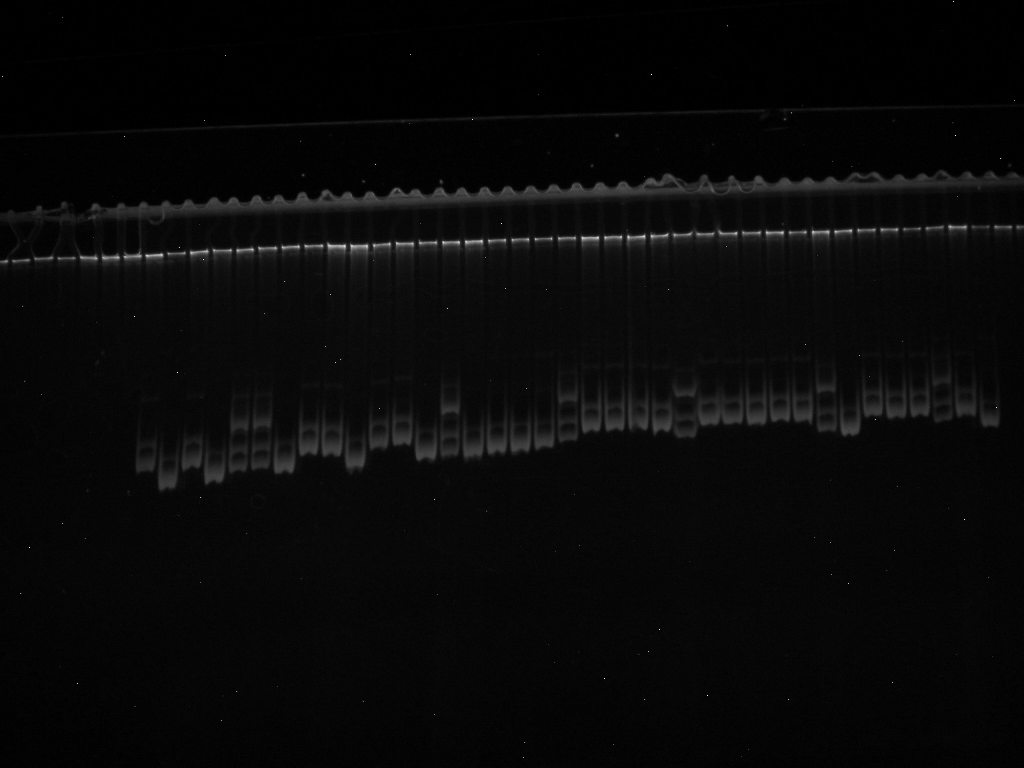


Note: This gel image was used to prepare Fig. 1C in the context of the manuscript.
